# Cellular Uptake of Gold Nanorods in Breast Cancer Cell Lines

**DOI:** 10.3390/nano12060937

**Published:** 2022-03-12

**Authors:** Bryan E. White, Molly K. White, Zeid A. Nima Alsudani, Fumiya Watanabe, Alexandru S. Biris, Nawab Ali

**Affiliations:** 1 Department of Biology, Donaghey College of Science, Technology, Engineering, and Mathematics, University of Arkansas at Little Rock, Little Rock, AR 72204, USA; nali@ualr.edu; 2 Center for Integrative Nanotechnology Sciences, University of Arkansas at Little Rock, Little Rock, AR 72204, USA; mksirigiri@ualr.edu (M.K.W.); zanima@ualr.edu (Z.A.N.A.); fxwatanabe@ualr.edu (F.W.); asbiris@ualr.edu (A.S.B.)

**Keywords:** nanomaterials, cellular uptake, bare gold nanorods, breast cancer cell lines, nanotechnology

## Abstract

Nanosized materials have been proposed for a wide range of biomedical applications, given their unique characteristics. However, how these nanomaterials interact with cells and tissues, as well as how they bio-distribute in organisms, is still under investigation. Differences such as the nanoparticle size, shape, and surface chemistry affect the basic mechanisms of cellular uptake and responses, which, in turn, affects the nanoparticles’ applicability for biomedical applications. Thus, it is vital to determine how a specific nanoparticle interacts with cells of interest before extensive *in vivo* applications are performed. Here, we delineate the uptake mechanism and localization of gold nanorods in SKBR-3 and MCF-7 breast cancer cell lines. Our results show both differences and similarities in the nanorod–cell interactions of the two cell lines. We accurately quantified the cellular uptake of gold nanorods in SKBR-3 and MCF-7 using inductively coupled plasma mass spectrometry (ICP-MS). We found that both cell types use macropinocytosis to internalize bare nanorods that aggregate and associate with the cell membrane. In addition, we were able to qualitatively track and show intracellular nanoparticle localization using transmission electron microscopy. The results of this study will be invaluable for the successful development of novel and “smart” nanodrugs based on gold nano-structural delivery vehicles, which heavily depend on their complex interactions with single cells.

## 1. Introduction

Nanoparticles are of extreme importance in many biological applications, including fluorescent biological labeling, bio-imaging, bio-sensing, gene/drug delivery, detection of pathogens, tissue engineering, and complex cancer therapy [1]. In addition, the interdisciplinary field of bioinformatics has biological applications in nanotechnology, which has opened new doors for the development of more nanomaterials [2,3,4,5]. However, it is well known that cells do not interact with all nanoparticles in the same way. Differences such as nanoparticle size, shape, and surface chemistry affect cellular uptake and response, and, thus, the nanoparticle’s usability for specific biological applications [6,7]. Determining how a certain type of nanoparticle interacts with specific cells is vital for determining the former’s usefulness and cellular impact.

This study aimed to quantify cellular uptake and investigate the endocytosis mechanics of bare gold nanorods in SKBR-3 and MCF-7 cell lines. These two cell lines have been used extensively as model cell lines for a variety of breast cancer treatment studies. Furthermore, these cell lines represent HER2-positive and HER2-negative breast cancer models, respectively. While many studies involving the cellular uptake of gold nanoparticles in cancer cells have been published [8,9,10], an extensive study comparing the cellular uptake of gold nanorods by SKBR-3 and MCF-7 has not been reported, to the best of our knowledge. When studying the uptake of nanorods by breast cancer cells, it is crucial to know the quantity of particles taken up at a specific concentration and time. It is also important to know if the nanorods are being internalized or binding to the surface of the cells. Finally, it is necessary to determine the endocytosis mechanism and the localization of the nanorods once internalized. This work is both significant to and necessary for designing nanomaterials and studying targeted drug delivery in breast cancer using nanomaterials and nanotechnology. Therefore it adds beneficial knowledge to the clinical arena.

Inductively coupled plasma mass spectrometry (ICP-MS) is generally considered a reliable approach for the quantification of nanoparticles’ interactions with cells [11,12], but it cannot indicate their corresponding location, or determine whether particles have been internalized or bound to the cell surface. Transmission electron microscopy (TEM) is a great analytical technique for confirming internalization and showing localization [13,14], but it is not ideal for quantification or as a standalone method to determine the endocytosis mechanism. Therefore, we used ICP-MS and TEM together to study and compare the quantification, internalization, localization, and uptake mechanism of the two different cell types. Our approach involved the use of TEM to determine cellular internalization at different time points and concentrations. TEM was also used to determine the localization of the nanorods once internalized. ICP-MS was used to quantify cellular uptake and delineate the endocytosis mechanism via known endocytosis inhibitors. This approach has been utilized in previous cellular uptake studies [15].

## 2. Materials and Methods

### 2.1. Nanorod Synthesis

Gold nanorods (AuNRs) were synthesized using the silver ion-assisted, seed-mediated method reported previously [16,17]. Briefly, the seed solution was prepared by combining 5 mL of cetyl trimthylammonium bromide (CTAB) solution (0.2 M) with 5 mL of HAuCl4 (0.0005 M), after which 600 mL of NaBH4 (0.01 M) was added and stirred for two minutes. To prepare gold nanorods with an aspect ratio of around 3, CTAB (5 mL, 0.2 M) was combined with 150 mL of silver nitrate solution (0.004 M), then 5 mL of HAuCl4 (0.001 M) was added and mixed. Next, 70 mL of ascorbic acid (0.0788 M) was combined with the solution, followed by 12 mL of seed solution. The combined solution was maintained at 30 °C for 40 min and not stirred. CTAB was taken out by washing the prepared nanorods repeatedly with a 1:1 mixture of deionized (DI) water and absolute ethanol, using centrifugation at 1000 rpm for 30 min each time. Finally, the solution was decanted, and the nanorods were redispersed with the DI + EtOH mixture via bath sonication. More than 200 nanorods were measured using ImageJ software (Bethesda, MA, USA) for the particle analysis function. The average length and diameter for the majority of the nanorods were determined to be around 36.00 nm and 12.00 nm, respectively.

### 2.2. Cell Culture

Two human breast cancer cell lines were used, SKBR-3 and MCF-7, obtained from the American Type Culture Collection (ATCC). SKBR-3 cells were cultured in McCoy’s 5A medium (Manassas, VA, USA) with 2 mM L-glutamine, and supplemented with 10% fetal bovine serum (FBS) and 1% penicillin and streptomycin in T-75 cell culture flasks. The passage number for all experiments was kept at less than 12. MCF-7 cells were cultured in Eagle’s Minimum Essential Medium (Manassas, VA, USA) supplemented with 10% FBS and 1% penicillin and streptomycin in T-75 cell culture flasks. The passage number for all experiments was kept at less than 12. SKBR-3 and MCF-7 cells were incubated at 37 °C in a humidified atmosphere in a 5% CO_2_ incubator. The cells were passaged according to the recommendations of ATCC.

### 2.3. Cytotoxicity

The MTT method was used to determine the viability of SKBR-3 and MCF-7 cells treated with different concentrations of AuNRs. Normal human fibroblast cells (BJ1 cell lines) were previously tested in our laboratory (not shown in this study), under similar conditions, and showed no cytotoxicity. The MTT method has been described previously [18,19]. Cells were seeded in 96-well plates at a density of 1.5 × 10^4^ cells per well, and were allowed to attach overnight in a CO_2_ incubator. Cells were then treated with 0, 5, 25, and 75 μg/mL of AuNRs for 24 and 48 h. The cells were then washed three times with PBS to remove the unbound gold nanoparticles. The MTT reagent was added 2 h before the termination of the experiment, and the optical density was acquired at 570 nm. The nanoparticle concentration and timeframe were determined based on cell viability below 70%.

### 2.4. Transmission Electron Microscopy

TEM samples were prepared according to a previously reported method [20]. SKBR-3 and MCF-7 cells were seeded in 6-well plates at a density of 0.5 × 10^6^ cells per well. After seeding, the cells adhered overnight in a tissue culture incubator at 37 °C and 5% CO_2_. The next day, the cells were treated with 5, 25, and 75 μg/mL of AuNRs for 1, 4, 24, and 48 h. Cells were washed three times with 1X phosphate-buffered saline and then prepared for TEM analysis. The cells were fixed with 2.5% glutaraldehyde in 0.1 M sodium cacodylate buffer at 4 °C for 20 min. The cells were then washed for a minimum of three times, 5 min each time, with 0.1 M sodium cacodylate buffer. After washing, the samples were post-fixed using 1% osmium tetroxide and 0.8% potassium ferricyanide in 0.1 M sodium cacodylate buffer for 1 h in the dark at room temperature. Post fixation, the cells were washed three times with 0.1 M sodium cacodylate buffer and stained with 1% tannic acid for 20 min. The cells were then stained with 0.5% uranyl acetate for 1 h at room temperature in the dark. After staining, the cell samples were dehydrated using increasing concentrations of absolute ethanol, followed by embedding in epoxy resin. Then, 50 nm slices were prepared with a diamond knife using a microtome, placed on copper grids, and post-stained prior to TEM imaging.

### 2.5. Inductively Coupled Plasma Mass Spectrometry

ICP-MS samples were prepared using the method described by Thermo Fisher Scientific (Memphis, TN, USA) and the Center for Applied Isotopes Studies (Fayetteville, USA) [21]. SKBR-3 and MCF-7 cells were seeded separately in 6-well plates at a density of 0.5 × 10^6^ cells per well. The cells were left to adhere overnight in a tissue culture incubator at 37 °C and 5% CO_2_. The next day, cells were treated with 0, 5, 25, and 75 μg/mL of AuNRs, and incubated for 1, 4, 24, and 48 h. Additional cells were pretreated with chlorpromazine and sucrose for one hour to inhibit clathrin-mediated endocytosis and macropinocytosis, respectively, then treated with 25 μg/mL of AuNRs for 24 h. Cells were washed and collected via trypsinization and centrifugation. Cell pellets, along with any AuNRs, were then dissolved using concentrated aqua regia at 80 °C. Next, any residual organic material was removed using hydrogen peroxide. Gold standards of different concentrations were prepared, and samples and standards were subjected to ICP-MS analysis with the Stable Isotopes Laboratory (University of Arkansas, Fayetteville, AR, USA). Measurements of pg/cell are based on normalization data given by the number of cells harvested at the time of the assay.

## 3. Results

### 3.1. Nanomaterial Characteristics

**Figure 1:**
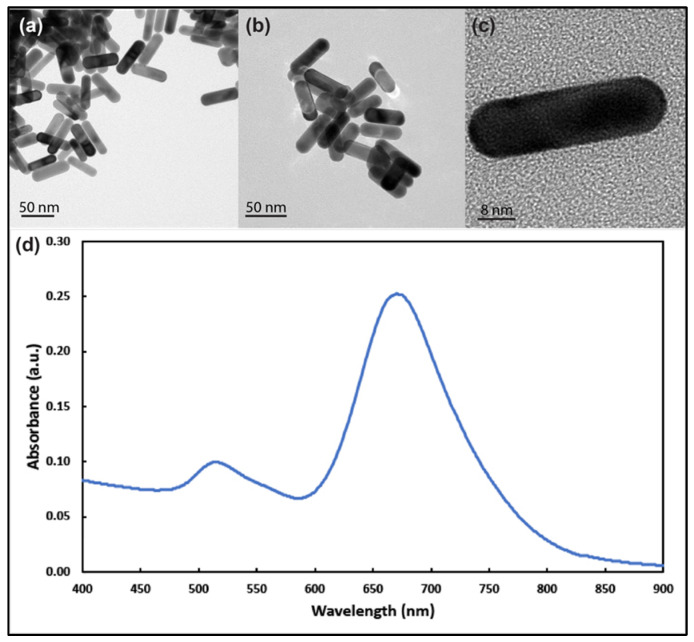
Transmission electron microscope images of gold nanorods with aspect ratio around 3 at different magnifications (**a**–**c**). (**d**) UV–visible spectrum analysis of the prepared gold nanorods showing longitudinal and transverse plasmons around 690 nm and 514 nm, respectively.

TEM images (Figure 1a–c) revealed that the majority of the AuNRs’ length and diameter were approximately 36 nm and 12 nm (some slightly longer), respectively, with longitudinal plasmon resonance at around 680 nm and transverse plasmon resonance at around 514 nm (Figure 1d). The zeta-potential average (n = 3) value for the prepared nanorods was −47.18 ± 4.26 mV.

### 3.2. Cytotoxicity

**Figure 2:**
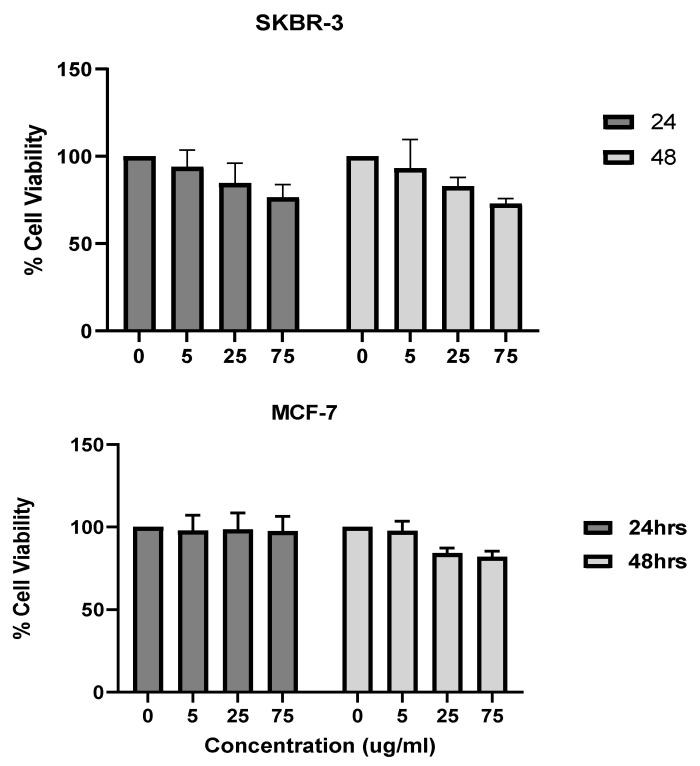
Cytotoxicity analysis (MTT method) of gold nanorods in concentrations ranging from 0 to 75 μg/mL at 24 h and 48 h in SKBR-3 cells (**top**) and MCF-7 cells (**bottom**). The SKBR-3 cells began to show a decrease in viability at 75 μg/mL and 48 h of exposure, while MCF-7 cells showed no decrease in cell viability. No statistically significant difference in cell viability was observed. The values shown are averages with standard errors, determined from at least three independent experiments, each conducted in triplicate.

The 3-[4,5-dimethylthiazole-2-yl]-2,5-diphenyltetrazolium bromide method, or MTT method, uses the conversion of MTT into formazan crystals that occurs in living cells. This process assesses mitochondrial activity. Due to the relationship between the total mitochondrial activity and the number of viable cells, this method is widely used to assess the cytotoxicity of drugs on cell lines or primary patient cells [22]. The SKBR-3 cells were more sensitive than the MCF-7 cells to nanorod exposure at higher concentrations and longer exposure times. The SKBR-3 cells dropped in viability at 48 h of exposure and 75 μg/mL of AuNRs. In contrast, the MCF-7 cells did not show any drop in cell viability (Figure 2). It is important to note that the viability did not drop to levels that would indicate toxicity in the SKBR-3 cells. The drop in SKBR-3 cell viability is due to the SKBR-3 cells’ sensitivity to the higher concentration (75 μg/mL) and longer exposure time (48 h). Research has shown that differences in gold nanoparticle shapes and sizes can result in differences in cell viability and toxicity [23]. Here, we observe that two different breast cancer cell lines respond somehow differently when exposed to the same gold nanorods, in terms of sensitivity. 

### 3.3. Quantification of Cellular Uptake Using ICP-MS

**Figure 3:**
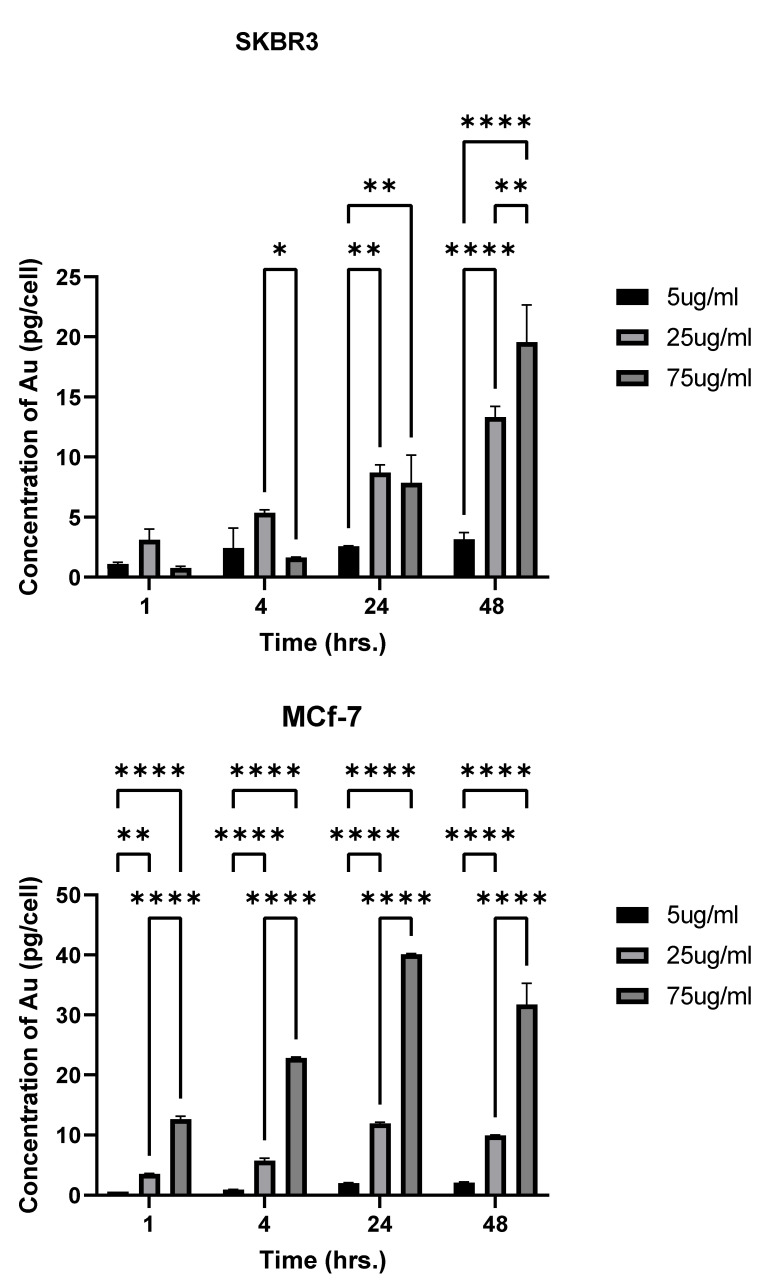
Uptake differences based on concentration: cellular uptake of AuNRs in SKBR-3 (**top**) and MCF-7 (**bottom**) cells at different time points, as analyzed by inductively coupled plasma mass spectrometry. Statistical differences between time points in a single concentration were determined based on *p* < 0.05. No differences were observed between concentrations at 1 h in SKBR-3. At 4 h with the 75 μg/mL concentration, there was not much change in accumulation. Differences were observed in MCF-7 at 1 h with all concentrations of AuNRs. MCF-7 also showed a difference between all concentrations at 4 h and 24 h, but this was not the case in SKBR-3. At 48 h, there was a significant difference in cellular uptake between all concentrations in both cell types (* indicates a p-value of less than 0.05, ** less than 0.005, **** less than 0.00005 etc.).

Differences in cellular uptake by the two cell lines were observed. In SKBR-3 cells, no statistical differences were observed in cellular uptake between nanorod concentrations at 1 h of exposure. At 4 h of exposure, differences were noted between the 25 and 75 μg/mL concentrations, with a higher quantity being taken up from the 25 μg/mL concentration. However, the MCF-7 cells did show a difference in cellular uptake between 1 and 4 h, with 75 μg/mL AuNR-treated cells taking up significantly more nanorods than the 5 and 25 μg/mL-treated cells. MCF-7 cellular uptake also differed between all the concentrations at 4 and 24 h, but this was not the case for SKBR-3. At 48 h of exposure, there was a significant difference in cellular uptake between all the concentrations in both cell types (Figure 3).

Most importantly, with respect to cellular uptake, when comparing the two cell lines, we observed a greater amount of nanorods taken up in SKBR-3 cells at 48 h of exposure, and concentrations of 5 and 25 μg/mL. Interestingly, this trend changed with a AuNR dose of 75 μg/mL, at which point MCF-7 showed greater nanorod uptake at all time points, except 48 h. At 48 h, there was no longer a difference in uptake by the two cell types.

### 3.4. Internalization Study of AuNRs Using TEM

**Figure 4:**
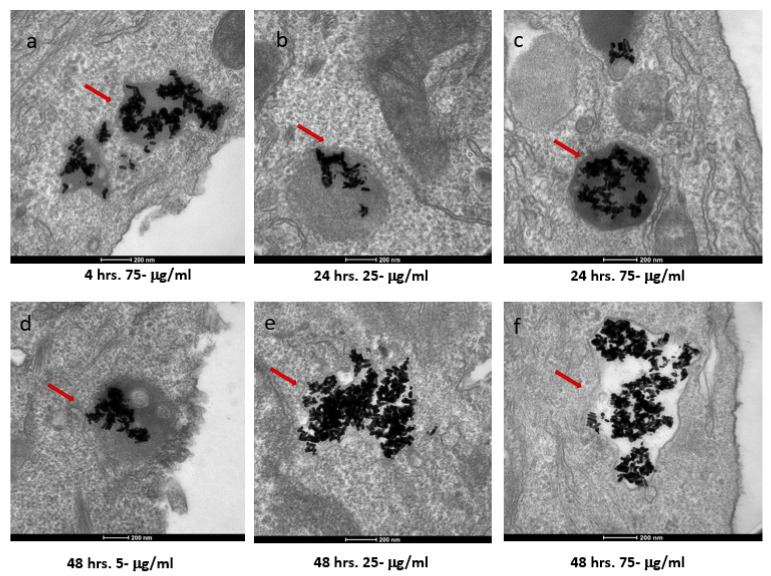
TEM images of AuNRs in SKBR-3 cells at different time points and concentrations. SKBR-3 cells were treated with 5, 25, and 75 μg/mL of AuNRs for 1, 4, 24, and 48 h. Internalization of nanorods was observed starting at 4 h incubation with 75 μg/mL (**a**). After 24 h of incubation, internalization was observed with the 25 μg/mL (**b**) and 75 μg/mL concentrations (**c**). For cells incubated for 48 h, we observed internalization with 5, 25, and 75 μg/mL concentrations (**d**, **e**, and **f**, respectively). Red arrows point to gold nanorods localized in SKBR-3 cells. Electron photomicrographs shown are representative of at least three independent experiments with similar conditions.

**Figure 5:**
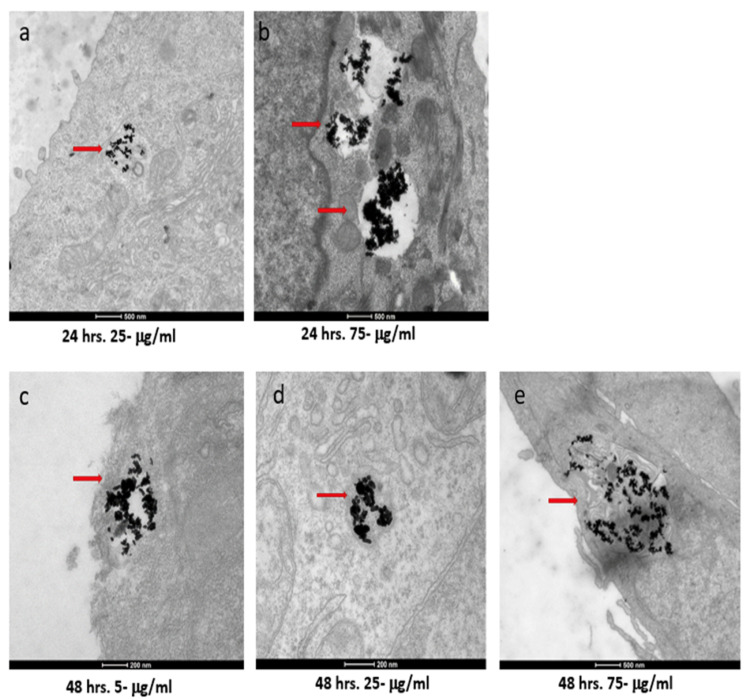
TEM images of AuNRs in MCF-7 cells at different time points and concentrations. MCF-7 cells were treated with 5, 25, and 75 μg/mL of AuNRs for 1, 4, 24, and 48 h. Internalization of nanorods was observed starting at 24 h with 25 and 75 μg/mL concentrations ((**a**) and (**b**), respectively). For cells incubated for 48 h, we observed internalization with 5, 25, and 75 μg/mL concentrations ((**c**), (**d**), and (**e**), respectively). Red arrows point to gold nanorods localized in MCF-7 cells. Electron photomicrographs shown are representative of at least three independent experiments with similar conditions.

The two cell types exhibited both similar and contrasting internalization behaviors. Internalization by the SKBR-3 cells was observed by 48 h with all three concentrations of nanorods. Indeed, at just 4 h, internalization of the 75 μg/mL concentration by the SKBR-3 cells was observed, as was a heavy interaction with the membrane by the 25 μg/mL concentration; by 24 h, internalization of both the 25 and 75 μg/mL concentrations was observed. At one hour of exposure, nanorods from the 25 and 75 μg/mL concentrations were observed interacting with the cell membrane. No internalization was observed with the 5 μg/mL treatment after 1, 4, or 24 h of exposure (Figure 4). On the other hand, the MCF-7 cells also internalized nanorods by 48 h for all three concentrations, and by 24 h for 25 and 75 μg/mL, but at 1 and 4 h, no internalization was observed (Figure 5).

### 3.5. Mechanism of Cellular Uptake

**Figure 6:**
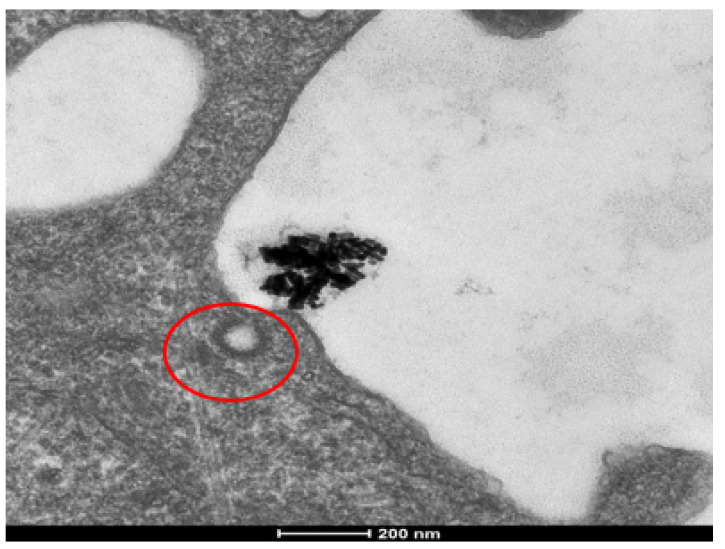
Cellular uptake analysis of AuNRs in SKBR-3 cells using TEM. To confirm internalization and the mechanism of endocytosis, clathrin pits were analyzed (pit circled in red). Due to the aggregation of AuNRs, the clumps were too large for receptor-mediated endocytosis and no AuNRs were observed to be localized in coated pits or vesicles. The electron photomicrograph shown is representative of at least three independent experiments with similar conditions. The same trend was observed in MCF-7 cells.

Our initial hypothesis concerning the mechanism of cellular uptake was that AuNRs would be endocytosed via the receptor-mediated method. Given the size and shape of AuNRs, research has shown this to be the most likely mechanism [24]. Using TEM, we analyzed clathrin pits to confirm receptor-mediated endocytosis, and found that none of the observed clathrin-coated pits contained nanorods (Figure 6). Further investigation showed that the size of the nanorod aggregates made receptor-mediated endocytosis an unlikely mechanism, as this process is for much smaller cargo. By measuring the cellular organelles and internal structures housing the internalized nanorods, and by analyzing the structural membranes, we concluded that the nanorods were being endocytosed via the macropinocytosis mechanism.

**Figure 7:**
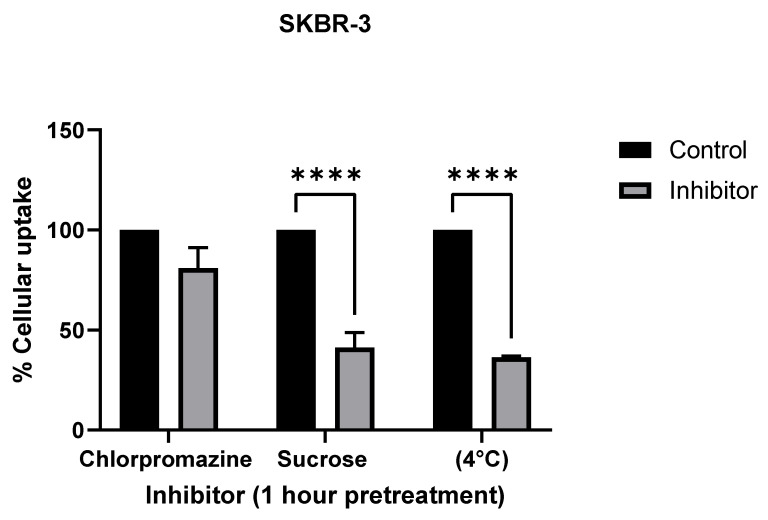

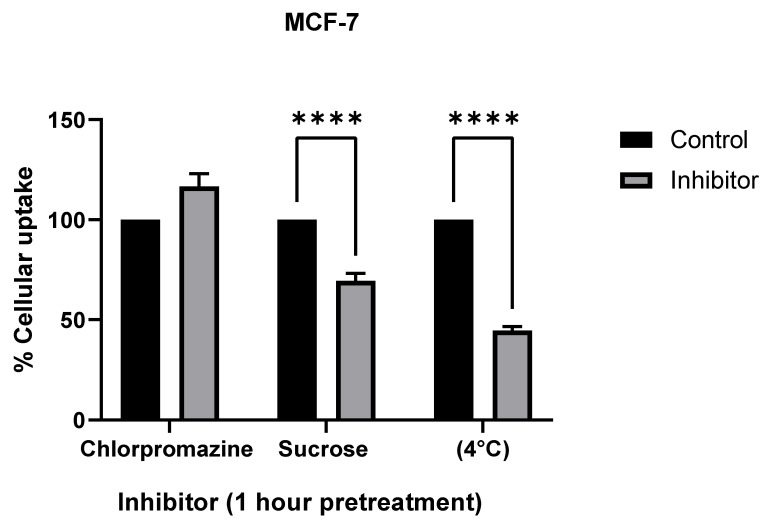
Comparison of endocytosis inhibitor treatments (chlorpromazine, sucrose, and temperature) to determine cellular uptake mechanism in SKBR-3 (**top**) and MCF-7 (**bottom**) cells. Both cell lines were used for cellular uptake of gold nanorods. Chlorpromazine is a known inhibitor for clathrin-mediated endocytosis, and sucrose is an inhibitor of both clathrin-mediated endocytosis and fluid phase macropinocytosis. Treatments at 4 °C were used as a positive control for inhibiting endocytosis. The values shown are averages with standard errors, determined from at least three independent experiments, each conducted in triplicate. (**** less than 0.00005 etc.).

To further confirm the mechanism of cellular uptake, we inhibited known mechanisms of endocytosis using the following inhibitors: chlorpromazine, an inhibitor for clathrin-mediated endocytosis (CME); hypertonic sucrose, which inhibits both CME and fluid phase micropinocytosis; reduced temperature (4 °C), which inhibits all endocytosis [25] (Figure 7). ICP-MS analysis showed a significant decrease in AuNR uptake with sucrose and low-temperature treatments in both SKBR-3 and MCF-7 cell lines.

### 3.6. Subcellular Localization

**Figure 8:**
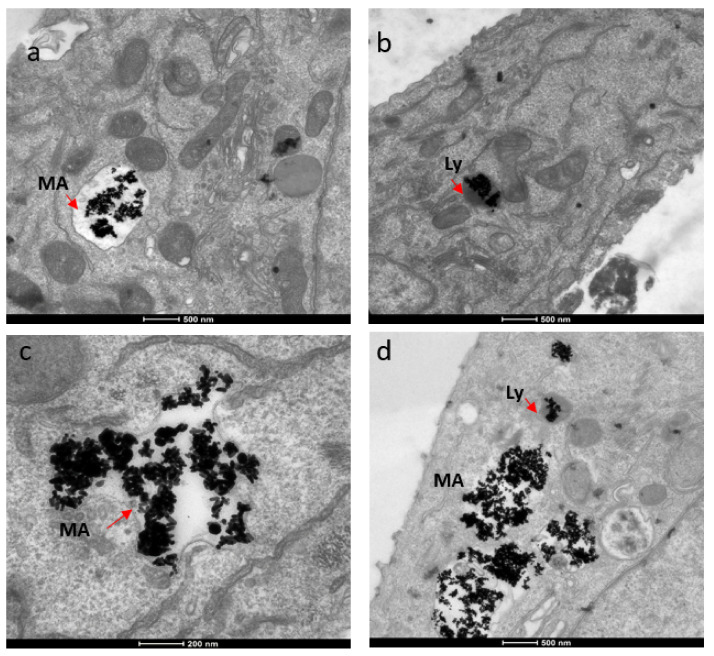
TEM images showing localization of gold nanorods in macropinosomes and lysosomes in both SKBR-3 and MCF-7 cell lines. AuNR localization in (**a**) macropinosomes (MA) and (**b**) lysosomes (Ly) of SKBR-3 cells. (**c**) Macropinosomes (MA) containing gold nanorods in MCF-7 and (**d**) lysosomes (Ly) containing gold nanorods in MCF-7. Red arrows point out macropinosomes and lysosomes containing gold nanorods in each image. Electron photomicrographs shown are representative of at least three independent experiments with similar conditions.

For subcellular localization, we used TEM techniques. The localization of AuNRs was rather similar in both breast cancer cell types. Huge clusters of gold nanorods were observed in membrane-bound intracellular structures, ranging in size from 1.3 to 2.7 micrometers (Figure 8). Given the size of the structure and the membrane type, these structures were most likely macropinosomes. Cargo the size of the clumped aggregates would likely be taken up via macropinocytosis, rather than a receptor-mediated method, such as clathrin-mediated endocytosis [24]. Macropinosomes range in size from 100 nm to 5 micrometers [26]. Once internalized, the macropinosome-containing gold nanorods most likely interact with lysosomes, possibly forming macropino-lysosomes. This is based on normal macropinocytosis trafficking behavior. AuNRs were also observed in lysosomes (Figure 8). Lysosomes generally range in size from 50 to 500 nm [27], but display much variation in size and shape, which results from the different types of materials taken up for digestion [28].

## 4. Discussion

When developing nanomaterials for the purpose of drug delivery in cancer studies and other applications, it is important and necessary to evaluate the interactions of target cells and tissues with the nanomaterials. Using bare nanorods in studies such as these can help us understand how changes made in modifying the nanomaterial’s surface for drug delivery functionalization affects the particle/cell interaction, in terms of cytotoxicity, cellular uptake quantity, and the mechanisms of endocytosis. Understanding the cellular uptake mechanisms and how particles accumulate in their targets before and after surface modification is a necessary and significant part of this process.

For clinical purposes, it will be necessary to define and establish the proper dosages in the case of using this technology for breast cancer drug delivery and functionalizing the nanorods for active targeting. It is also vital to understand exactly how the particles interact with the cells to properly design and equip these nanomaterials for the functionalization of drug delivery. Current FDA-approved nanodrugs, for example, have well-defined functionality, in terms of how they achieve specific targeting and delivery of cancer therapy [29].

This research provides a reliable technique to study cellular uptake mechanisms in vitro, and provides valuable information about the interactions of bare AuNRs in two different types of breast cancer cell lines commonly used as in vitro breast cancer models. This information will be valuable for assessing the efficacy of AuNRs functionalized for active targeting and drug delivery in further studies using the same breast cancer cell types.

Currently, research has shown aspects of cellular uptake in breast cancer cell lines [15], but a study that simultaneously compares the similarities and differences in HER2-positive (SKBR3) and HER2-negative (MCF7) cytotoxicity, cellular uptake, distribution, and endocytosis mechanisms of bare gold nanorods is not available in the literature to compare with our results.

Studying the cellular uptake process can be accurately accomplished by a combination of ICP-MS and TEM. Using these techniques with bare AuNRs, we found that two different types of breast cancer cells (SKBR-3 and MCF-7) respond in similar ways, but also show some differences. The similarities observed in the two cell lines were that both cell types, to a great extent, use the macropinocytosis mechanism to internalize gold nanoparticles, which aggregate and associate with the cell membrane. In addition, we were able to track the intracellular localization, finding that nanoparticles localized in macropinosomes and lysosomes. The differences observed between the two cell types included sensitivity, in terms of the effects on cell viability. We also observed differences in cellular uptake quantity at different time points and concentrations. These differences suggest that varying doses may be necessary in future targeting and drug delivery applications. The similarities, as well as the differences, observed here are likely due to the differences in cell type, given that all the other experimental conditions were the same.

Further studies will show how these same processes compare in the case of AuNRs surface modified and functionalized for targeting and drug delivery in these two breast cancer cell types.

## 5. Conclusions

In this research, we studied how two types of cancer cells that are common in cancer research integrate and uptake AuNRs with an aspect ratio of about three. Our results indicate that slight variations in cell types can change the way cells interact with gold nanorods. Thus, it is crucial to study each model independently to understand the way specific cells respond to a nanoparticle of interest. It is vital to analyze toxicity, quantify cellular uptake, and determine the endocytosis mechanism, localization, trafficking behavior, and even exocytosis with each new model. Understanding these processes is essential for the successful development of novel and “smart” nanodrugs that utilize nano-structural delivery vehicles, as such technologies heavily depend on their complex interactions with single cells.
